# Translational Bioinformatics and Computational Systems Medicine

**DOI:** 10.1155/2013/375641

**Published:** 2013-05-19

**Authors:** Bairong Shen, Hong-Bin Shen, Tianhai Tian, Qiang Lü, Guang Hu

**Affiliations:** ^1^Center for Systems Biology, Soochow University, P.O. Box 206, Suzhou 215006, China; ^2^Department of Automation, Shanghai Jiao Tong University, No. 800 Dongchuan Road, Shanghai 200240, China; ^3^School of Mathematical Sciences, Monash University, Melbourne, VIC 3800, Australia; ^4^School of Computer Science and Technologies, Soochow University, Suzhou 215006, China

This special issue is dedicated to Translational bioinformatics and Computational Systems Medicine. Translational biomedical informatics is a rapidly emerging discipline to integrate data from medical research, biotechnologies, and electronic medical records, and computational systems medicine is to apply computational and systems biology approaches to solve complex problems in medical research, aiming to improve the diagnosis, prognosis, and treatment of complex diseases. It is also well known that their development needs an integration of mathematical models, statistical methods, and computer algorithms.

 Complex diseases such as cancers are coursed by a combination of genetic, environmental, and lifestyle factors, and thus the research of complex diseases at a system level like gene sets, pathway level, or static/dynamic network is a necessity. T. Holden et al. present an exploratory bioinformatics study of long noncoding RNA in Alzheimer's disease. The authors also discuss the model for drug development based on fractal dimension and entropy correlation in this study consistent with a zebrafish model and a mouse model. M. Ding et al. explore the genome-wide chromatin localization of Estrogen receptor-DNA binding regions by analyzing ChIP-Seq data from MCF-7 breast cancer cell line. It reveals novel Estrogen receptor-regulated genes pathways for further experimental validation. Y.-w. Lv et al. perform gene ontology category and pathways analysis for relationships among statistically significant genes in Kawasaki disease. The importance of compelling immune pathway of NF-AT signal and leukocyte interactions combined with another transcription factor NF-*κ*B in the pathogenesis of KD is investigated by network analysis. J. Chen et al. perform two case studies on colorectal and prostate cancer microarray datasets to proof two hypotheses that (1) the expression signatures of different cancer microarray datasets are more similar at pathway level than at gene level; (2) the comparability of the cancer molecular mechanisms of different individuals is related to their genetic similarities.

The concept of entropy suggests that systems naturally progress from order to disorder. Entropy-based methods provide a novel insight into understanding many phenomena in biological systems. V. Oswal et al. present an automated entropy-based thresholding system for segmentation and quantification of cell nuclei from histologically stained images. The contributions to the application of this entropy-based system to detect cancerous cell nuclei and observe overlapping cellular events occurring during wound healing process in the human body are also presented. H.-T. Wu et al. investigate the feasibility and sensitivity of a novel multiscale entropy index in detecting subtle vascular abnormalities in healthy and diabetic subjects. The authors further focus on four groups of subjects to discuss the application of multiscale entropy index.

The role of protein structures in understanding diseases becomes more and more important, due to the following two reasons. One is that there are a lot of disease-associated proteins that were discovered, while the other fact is that many diseases are believed to result from misfolded proteins. Moreover, protein structures can be considered as complex systems, and thus network theory can be used to characterize and to analyze protein structures. J. Sun et al. present a contribution which focuses on the effect of edge definition of complex networks on protein structure identification. After the performance on 2847 proteins, the authors argue that the optimal cutoff value for constructing the protein structure networks is 5.0 Å. X. Jiao et al. improve the amino acid network for proteins by introducing similar weight and dissimilar weight. This work demonstrates that highly central residues of the amino acid network are highly correlated with the hot spots in disease-associated proteins.

Finally, two bioinformatics tools were also involved in this issue. H. Wu et al. contribute a bioinformatics tool called patGPCR, to predict the 3D structures of transmembrane helices of G-protein-coupled receptors. patGPCR, a parallelized multitemplate approach, extends a bundle-packing related energy function to RosettaMem energy, which improves the TM RMSD (root mean square deviation of the transmembrane helices) of the predicted models. N. Deng et al. contribute another platform, called crcTRP, for colorectal cancer. This server provides the translational research of colorectal cancer by providing various types of biomedical information, including clinical data, epidemiology data, individual omics data, and public omics data.

*Bairong Shen* *Hong-Bin Shen* *Tianhai Tian* *Qiang Lü* *Guang Hu*

